# The mesenchymal morphology of cells expressing the EML4–ALK V3 oncogene is dependent on phosphorylation of Eg5 by NEK7

**DOI:** 10.1016/j.jbc.2024.107144

**Published:** 2024-03-06

**Authors:** Sarah L. Pashley, Savvas Papageorgiou, Laura O'Regan, Giancarlo Barone, Susan W. Robinson, Kellie Lucken, Kees R. Straatman, Joan Roig, Andrew M. Fry

**Affiliations:** 1 Department of Molecular and Cell Biology, University of Leicester, Leicester, UK; 2 Advanced Imaging Facility, Core Biotechnology Services, University of Leicester, Leicester, UK; 3 Department of Cell & Developmental Biology, Molecular Biology Institute of Barcelona (IBMB-CSIC), Barcelona, Spain

**Keywords:** EML4–ALK, NEK7, NEK9, microtubules, Eg5, Eg5 inhibitors, NSCLC

## Abstract

Echinoderm microtubule-associated protein-like 4 (EML4)–anaplastic lymphoma kinase (ALK) oncogenic fusion proteins are found in approximately 5% of non–small cell lung cancers. Different EML4–ALK fusion variants exist with variant 3 (V3) being associated with a significantly higher risk than other common variants, such as variant 1 (V1). Patients with V3 respond less well to targeted ALK inhibitors, have accelerated rates of metastasis, and have poorer overall survival. A pathway has been described downstream of EML4–ALK V3 that is independent of ALK catalytic activity but dependent on the NEK9 and NEK7 kinases. It has been proposed that assembly of an EML4–ALK V3–NEK9–NEK7 complex on microtubules leads to cells developing a mesenchymal-like morphology and exhibiting enhanced migration. However, downstream targets of this complex remain unknown. Here, we show that the microtubule-based kinesin, Eg5, is recruited to interphase microtubules in cells expressing EML4–ALK V3, whereas chemical inhibition of Eg5 reverses the mesenchymal morphology of cells. Furthermore, we show that depletion of NEK7 interferes with Eg5 recruitment to microtubules in cells expressing EML4–ALK V3 and cell length is reduced, but this is reversed by coexpression of a phosphomimetic mutant of Eg5, in a site, S1033, phosphorylated by NEK7. Intriguingly, we also found that expression of Eg5-S1033D led to cells expressing EML4–ALK V1 adopting a more mesenchymal-like morphology. Together, we propose that Eg5 acts as a substrate of NEK7 in cells expressing EML4–ALK V3 and Eg5 phosphorylation promotes the mesenchymal morphology typical of these cells.

Lung cancer remains the leading cause of cancer-related mortality accounting for approximately 25% of all cancer deaths (1). Typically, lung cancers are classified as either non–small cell lung cancer (NSCLC) or small cell lung cancer with 85% being NSCLC. NSCLCs can be further categorized into adenocarcinomas, squamous cell carcinomas, or large cell carcinomas, depending on histological analysis (2, 3). The echinoderm microtubule-associated protein-like 4 (EML4)–anaplastic lymphoma kinase (ALK) fusion is a common genetic alteration estimated to exist in ∼5% of lung adenocarcinomas (4). EML4–ALK fusions have also been identified in other aggressive cancers, including breast, colorectal, and pancreatic cancers, albeit more rarely (5, 6, 7). EML4–ALK-positive (ALK+) lung cancers are treated with targeted ALK inhibitors as the current standard of care. However, these patients eventually relapse as their cancers inevitably develop resistance through secondary ALK mutations or activation of alternative bypass signaling pathways that drive oncogenic progression (8, 9, 10). Consequently, there is a clear need to better understand the mechanisms involved in EML4–ALK-driven cancers so that new therapies can be developed that are capable of overcoming this resistance.

While EML4–ALK was first discovered in lung cancer patients in 2007 (4), multiple variants of this fusion protein have since been identified that arise through different breakpoints in the *EML4* gene (11). All variants contain the same C-terminal fragment of ALK, including its cytoplasmic tyrosine kinase domain, but diverge in the amount of the EML4 N-terminal domain they encompass. The two most common variants, variant 1 (V1) and variant 3 (V3), collectively account for approximately 80% of ALK+ cancers (11, 12). However, there is a marked difference in the amount of EML4 protein present in these two variants (11). Wildtype EML4 consists of an N-terminal coiled coil that promotes trimerization followed by a basic unstructured region and a highly structured C-terminal TAPE domain containing a pair of seven-bladed β-propellers (13). Interestingly, both the coiled coil and basic region facilitate microtubule binding (14). EML4–ALK V1 encodes the full N terminus of EML4, with the coiled coil and basic region, and a truncated TAPE domain. EML4–ALK V3 on the other hand encodes only the N-terminal portion of EML4 and contains none of the TAPE domain (15). Importantly, EML4–ALK V3 is associated with reduced sensitivity to ALK inhibitors and worse outcomes in patients, including increased metastatic spread and lower survival rates (10).

In cultured cells, EML4–ALK V3, but not V1, localizes to interphase microtubules (14, 16). This finding seems at odds with both V1 and V3 containing the N-terminal region of EML4 that confers microtubule association. However, it has been found that certain structural elements of the partial TAPE domain present in V1 but not V3 interfere with microtubule localization (17). Importantly, cells expressing EML4–ALK V3 adopt a different morphology to those that express V1, with V3-expressing cells developing a more elongated shape with long cytoplasmic protrusions, reminiscent of mesenchymal cells. The development of a mesenchymal-like morphology enables cancer cells to undergo metastasis, one of the hallmarks of cancer (18). In contrast, cells expressing V1 adopt a morphology more reminiscent of normal lung epithelial cells. Cells expressing V3 also exhibit an enhanced rate of migration. These phenotypic changes in EML4–ALK V3 cells have been shown to depend on expression of the cell cycle–dependent kinases, NEK9 and NEK7, but not on ALK catalytic activity (16). Moreover, in cells expressing EML4–ALK V3, NEK9 and NEK7 are recruited to microtubules, where it is hypothesized that they assemble into a complex with EML4–ALK V3 leading to their constitutive activation (16). However, it remains unclear how this microtubule-associated EML4–ALK V3–NEK9–NEK7 complex leads to altered cell morphology and increased migration.

In cycling cells, NEK9, NEK7, and the closely related NEK6 kinase are normally activated at the G2/M transition with NEK9 upstream of NEK7 and NEK6 (19, 20). In mitosis, NEK6 is capable of phosphorylating the microtubule-based kinesin-5 motor, Eg5 (also known as KIF11) (21). Eg5 does not associate with interphase microtubules but is recruited to microtubules of the mitotic spindle primarily as a result of phosphorylation at T926 by CDK1 (22, 23). However, phosphorylation of Eg5 at S1033 by NEK6 also promotes recruitment of Eg5 to spindle poles facilitating their separation at the G2/M transition (21, 24). Further data suggest that NEK7 also phosphorylates Eg5 at the same residue as it was demonstrated using a phosphospecific antibody that S1033 phosphorylation was decreased following NEK7 depletion from cells (24). In addition, NEK7 depletion perturbed centrosome separation in over 25% of prophase cells (24).

Eg5 is a tetrameric molecule with opposing motor domains that crosslink and bundle adjacent microtubules. This enables Eg5 to slide antiparallel microtubules, pushing the centrosomes apart and establishing a bipolar spindle in mitosis (25, 26). Intriguingly, phosphorylation of Eg5 at S1033 has also been shown to promote dendrite extension in differentiating neurons (27). In this case, it appears that phosphorylation is catalyzed by NEK7 as depletion of NEK7, but not NEK6 or NEK9, reduced dendrite length. Subsequent transfection of a phosphomimetic Eg5-S1033D construct, but not wildtype or phosphonull Eg5-S1033A construct, rescued dendrite length confirming the importance of phosphorylation of Eg5 at this site. Moreover, treatment of neurons with an Eg5 rigor inhibitor, which locks Eg5 on microtubules, was found to promote microtubule stabilization (27). This suggests that Eg5 can be phosphorylated at the same site by either NEK6 or NEK7 in different cellular contexts. In addition to causing microtubule sliding, Eg5 has been shown to have microtubule polymerase activity (28, 29). Eg5 can track microtubule plus ends leading to the proposal that Eg5 might promote a kinked-to-straight transition when a tubulin heterodimer is added to a growing protofilament. This stabilizes the protofilament and permits microtubule polymerization (28, 29).

Here, we show that Eg5 is recruited to interphase microtubules in cells expressing EML4–ALK V3, but not V1, and that this is dependent on NEK7. Meanwhile, chemical inhibition of Eg5 prevents development of the mesenchymal cell morphology induced by EML4–ALK V3. Furthermore, experiments using phosphomimetic Eg5-S1033 constructs in cells expressing either EML4–ALK V1 or V3 support the hypothesis that these morphology changes are dependent on NEK7 phosphorylation of Eg5. As well as providing mechanistic insights on how EML4–ALK V3 alters cellular properties, these data raise the prospect that Eg5 inhibitors may be valuable alternative treatments for patients with EML4–ALK V3–driven cancers.

## Results

### Eg5 is recruited to interphase microtubules in EML4–ALK V3–expressing cells

**Figure 1:**
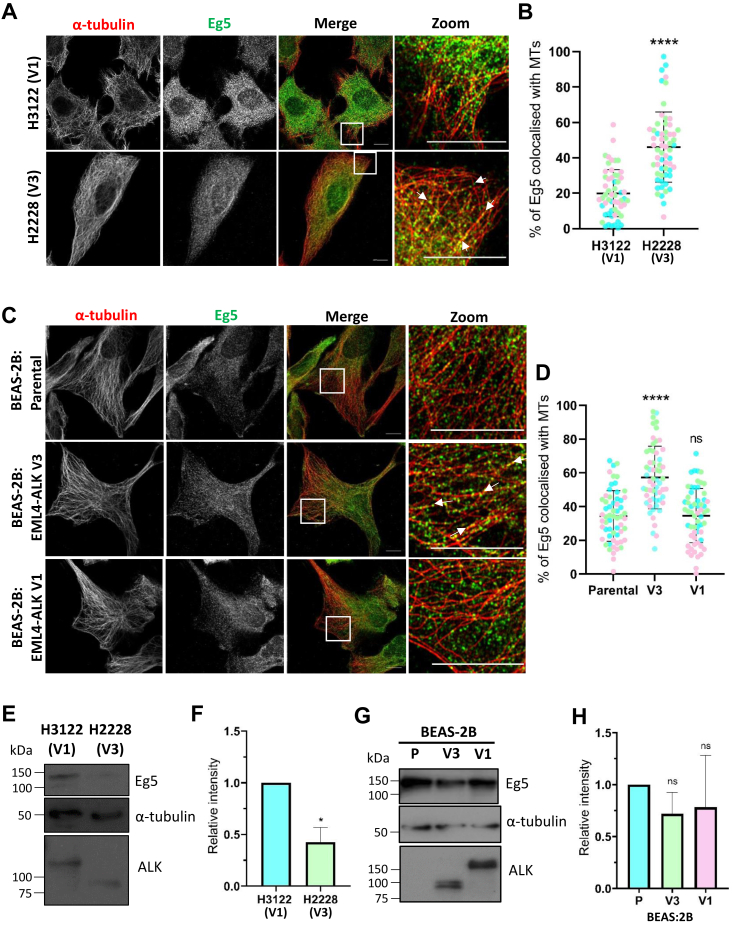
**Eg5 is recruited to interphase microtubules in EML4–ALK V3–expressing cells.***A*, ALK+ NSCLC patient cell lines, H3122 (V1) and H2228 (V3), were stained with antibodies against Eg5 (*green*) and α-tubulin (*red*), and single super-resolution optical sections were acquired. *White boxes* indicate zoom region. *B*, Imaris colocalization analysis was performed on images represented in *A*. Student’s unpaired *t* test was used to compare groups; ∗∗∗∗*p* < 0.0001. *C*, parental BEAS-2B or BEAS-2B stable cell lines induced to express EML4–ALK V1 or V3 as indicated were stained as described in *A*. *D*, Imaris colocalization analysis was performed on images represented in *C*. One-way ANOVA with post hoc Games–Howell testing was used to compare groups; ∗∗∗∗*p* < 0.0001. Where not significant (NS), *p* = 0.9963. In *A* and *C*, merge images are shown alongside magnified view (*zoom*) from the merge images. Scale bars represent 10 μm. *White arrows* indicate localization of Eg5 to microtubules. *B* and *D*, graphs represent individual data points from three independent experiments (one color per repeat); means and standard deviations are indicated. Significance is shown in *B* for H2228 (V3) compared with H3122 (V1) and in *D* for the induced V1 and V3 cells compared with parental BEAS-2B cells. *E*, lysates from ALK+ NSCLC patient cell lines, H3122 (V1) and H2228 (V3), were analyzed by Western blot using the antibodies indicated. *F*, quantification of Eg5 intensity and normalized against α-tubulin intensity from Western blots represented in *E*. Data are shown relative to Eg5 intensity in H3122 (V1) cells. ∗*p* < 0.05. *G*, lysates from parental BEAS-2B cells (P) or BEAS-2B cells induced to express EML4–ALK V3 or V1 were analyzed by Western blot using the antibodies indicated. *H*, quantification of Eg5 intensity and normalized against α-tubulin intensity from Western blots represented in *G*. Data are shown relative to Eg5 intensity in the parental cells. Statistical comparisons were made using Student's unpaired *t* test where NS, *p* = 0.5281 and *p* = 0.1422, respectively. Molecular weights are shown to the *left* of the blots in *E* and *G*. Blots and graphs are representative of three independent experiments. Graphs *F* and *H* show means and standard deviations. ALK, anaplastic lymphoma kinase; EML4, echinoderm microtubule-associated protein-like 4; NSCLC, non–small cell lung cancer.

Given that (i) EML4–ALK V3 binds to interphase microtubules where it recruits NEK9 and NEK7, (ii) Eg5 is a known substrate of NEK7, and (iii) NEK7 promotes recruitment of Eg5 to microtubules in neuronal cells, we wished to test the hypothesis that Eg5 is a downstream target of the EML4–ALK V3–NEK9–NEK7 complex. For this purpose, we began by analyzing the localization of Eg5 in cells expressing EML4–ALK V3 in the knowledge that Eg5 normally only associates with microtubules in mitosis and not interphase (22). First, lung cancer patient cell lines, H3122 and H2228, that express EML4–ALK V1 and V3, respectively, were examined. Cells were stained with antibodies against Eg5 and α-tubulin and analyzed by immunofluorescence microscopy. Strikingly, magnified views revealed that although Eg5 staining appeared punctate in both cell lines, these punctae were more often seen along the length of interphase microtubules in H2228 (V3) than H3122 (V1) cells (Fig. 1*A*). Consistent with this, colocalization analysis using Imaris software (Oxford Instruments) revealed a significantly greater than two-fold increased association of Eg5 with microtubules in H2228 (V3) than H3122 (V1) cells (Fig. 1*B*).

As these are cancer-derived cell lines, the H3122 (V1) and H2228 (V3) cells harbor additional genetic alterations to the EML4–ALK variants. Indeed, according to the COSMIC database, Eg5 contains an A282V missense mutation in H2228 (V3) cells that may alter its microtubule affinity. We therefore decided to test the distribution of Eg5 in a set of isogenic BEAS-2B bronchial epithelial cell lines that express EML4–ALK V1 or V3 after induction with doxycycline (30). Immunofluorescence microscopy again revealed a punctate staining pattern for Eg5 in BEAS-2B cell lines (Fig. 1*C*). In agreement with data obtained from the patient cell lines, quantification revealed that significantly more Eg5 was present on interphase microtubules in BEAS-2B:EML4–ALK V3 cells, as compared with either the parental BEAS-2B or BEAS-2B:EML4–ALK V1 cells that had similar and lower levels of colocalization of Eg5 with microtubules (Fig. 1*D*).

Eg5 expression is commonly upregulated in cancers (31, 32, 33, 34). Therefore, we tested whether Eg5 expression was altered upon expression of EML4–ALK V3. Lysates were generated from H3122 (V1) and H2228 (V3) cells and analyzed by Western blot to assess Eg5 protein levels. Interestingly, we found that Eg5 was substantially reduced in the H2228 (V3) compared with H3122 (V1) cell line (Fig. 1, *E* and *F*). However, this could be due to the missense mutation in Eg5 leading to either the protein or mRNA being less stable than the wildtype version. Eg5 expression was also assessed in the induced BEAS-2B cell lines as before. This time, we found no significant difference in Eg5 expression in the BEAS-2B cell lines upon induction of EML4–ALK V1 or V3 (Fig. 1, *G* and *H*). Taken together, these results clearly demonstrate an increased recruitment of Eg5 to microtubules in cells expressing EML4–ALK V3. This increase in recruitment was found despite the fact Eg5 expression was equal in the two inducible BEAS-2B cell lines and was in fact lower in the H2228 patient-derived cells that express V3 compared with H3122 cells that express V1. This indicates that the increase in Eg5–microtubule colocalization is not simply a result of higher Eg5 protein expression.

### Eg5 inhibitors reverse the mesenchymal morphology of cells expressing EML4–ALK V3

Following our observation that Eg5 is recruited to interphase microtubules in cells expressing EML4–ALK V3, we wished to know whether Eg5 motor activity contributes to their mesenchymal-like morphology. Chemical inhibitors of Eg5 typically fall into one of two classes dependent on where they bind the motor domain: loop 5 (L5) or rigor inhibitors. L5 inhibitors, such as STLC or filanesib, weaken the interaction between Eg5 and microtubules, whereas rigor inhibitors, such as BRD9876, tighten this interaction and stabilize microtubules (35). Inhibitors from both categories were used to inhibit Eg5 in BEAS-2B cells. Because of its role in promoting centrosome separation, inhibition of Eg5 with both classes of inhibitor should lead to mitotic arrest with monopolar spindles (23). Live cell imaging confirmed that these drugs worked as expected with a large proportion of BEAS-2B:EML4–ALK V1 and BEAS-2B:EML4–ALK V3 cells found to undergo mitotic arrest as determined by a sustained rounded morphology after exposure for 24 h (Fig. S1, *A*–*C*). Moreover, immunofluorescence microscopy staining for microtubules confirmed the presence of monopolar spindles in these mitotic cells (Fig. S1*D*).

**Figure 2:**
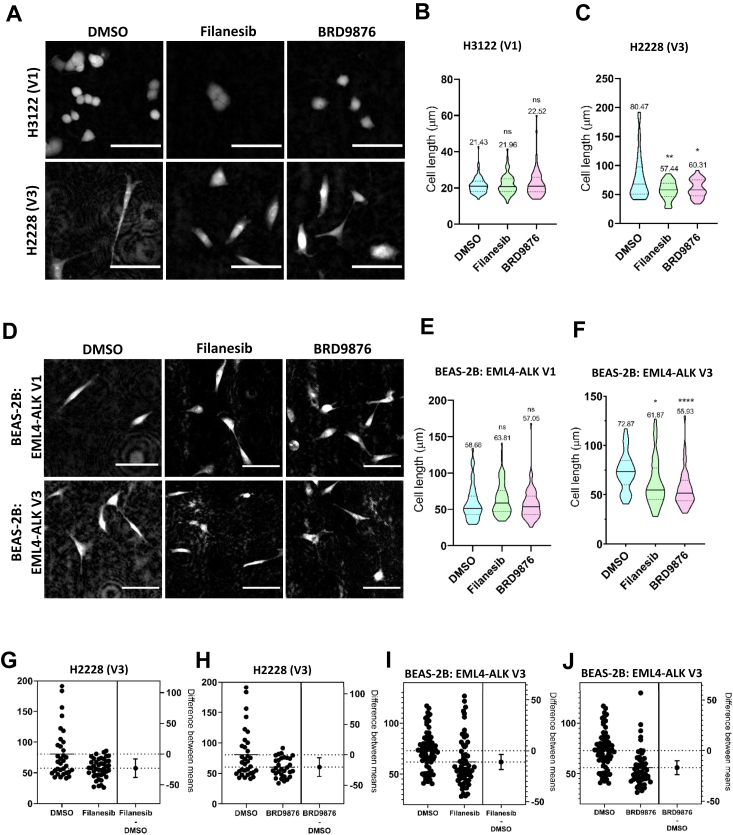
**Eg5 inhibitors reduce the length of cells expressing EML4–ALK V3.***A*, ALK+ NSCLC patient cell lines, H3122 (V1) and H2228 (V3), were embedded into collagen and treated with dimethyl sulfoxide (DMSO) alone, filanesib (100 nM), or BRD9876 (10 μM) for 24 h. Cells were then imaged by obtaining phase images using the LiveCyte 2 system. *B* and *C*, the violin plots indicate the length of H3122 (V1) (*B*) or H2228 (V3) (*C*) cells treated as in *A*. Statistical comparisons were performed either by one-way ANOVA with post hoc Games–Howell test for data in (*B*), which were not significant, *p* = 0.8096 and *p* = 0.5176, respectively, or by Dunnett’s T3 test for data in (*C*) where ∗∗*p* = 0.0069 and ∗*p* = 0.0219, respectively. *D*, induced BEAS-2B:EML4–ALK V1 and V3 were treated and stained as in *A*. Scale bars in *A* and *D* represent 100 μm. *E* and *F*, the violin plots indicate the length of BEAS-2B:EML4–ALK V1 (*E*) or BEAS-2B:EML4–ALK V3 (*F*) cells treated as in *D*. Statistical comparisons were performed by one-way ANOVA with post hoc Games–Howell test where in *E*, NS, *p* = 0.3932, and *p* = 0.9157, respectively, and in *F*, ∗*p* = 0.0125 and ∗∗∗∗*p* < 0.0001. Violin plots represent data from three independent experiments. The *solid center line* represents the median, *dotted lines* represent the quartiles, and means for each group are written above each plot. Significance is shown for each drug-treated sample compared with DMSO alone. *G*–*J*, estimation plots of the data shown in *B*, *C*, *E*, and *F*, respectively. ALK, anaplastic lymphoma kinase; EML4, echinoderm microtubule-associated protein-like 4; NSCLC, non–small cell lung cancer.

To measure morphology, cells were embedded in collagen to give a closer representation of a tumor microenvironment. In agreement with published data (16), we noted that induced BEAS-2B cells expressing EML4–ALK V3 were significantly longer than cells expressing EML4–ALK V1. Likewise, H2228 (V3) cells embedded in collagen were also significantly longer than H3122 (V1) cells. Embedded H3122 (V1) and H2228 (V3) cell lines were treated with Eg5 inhibitors for 1 h before being imaged. Cell length was then measured by drawing a line over the longest cell length in Fiji (36). Strikingly, quantitative imaging revealed that both filanesib and BRD9876 significantly reduced the length of H2228 (V3) cells but had no significant impact on the length of H3122 (V1) cells, which are a more rounded cell type even without drug treatment (Fig. 2, *A*–*C*). Similar experiments were undertaken with the isogenic BEAS-2B cell lines. EML4–ALK expression was induced for 48 h before cells were treated with the Eg5 inhibitors as before. Consistent with the data in the lung cancer patient cell lines, both filanesib and BRD9876 significantly reduced the length of BEAS-2B:EML4–ALK V3 cells but had no effect on the length of BEAS-2B:EML4–ALK V1 cells (Fig. 2, *D*–*F*). Estimation plots confirmed the statistical difference between the means for treated *versus* untreated H2228 (V3) and BEAS-2B:EML4–ALK V3 cells (Fig. 2, *G*–*J*). The fact that cell length was reduced equally by both drug treatments in both the patient-derived cells and inducible BEAS-2B cells strongly suggests that Eg5 must not only associate with microtubules but also be capable of presumably motor-dependent movement along microtubules in order to promote the mesenchymal-like morphology adopted by cells expressing EML4–ALK V3.

### NEK7 depletion reduces microtubule recruitment of Eg5 in cells expressing EML4–ALK V3

**Figure 3:**
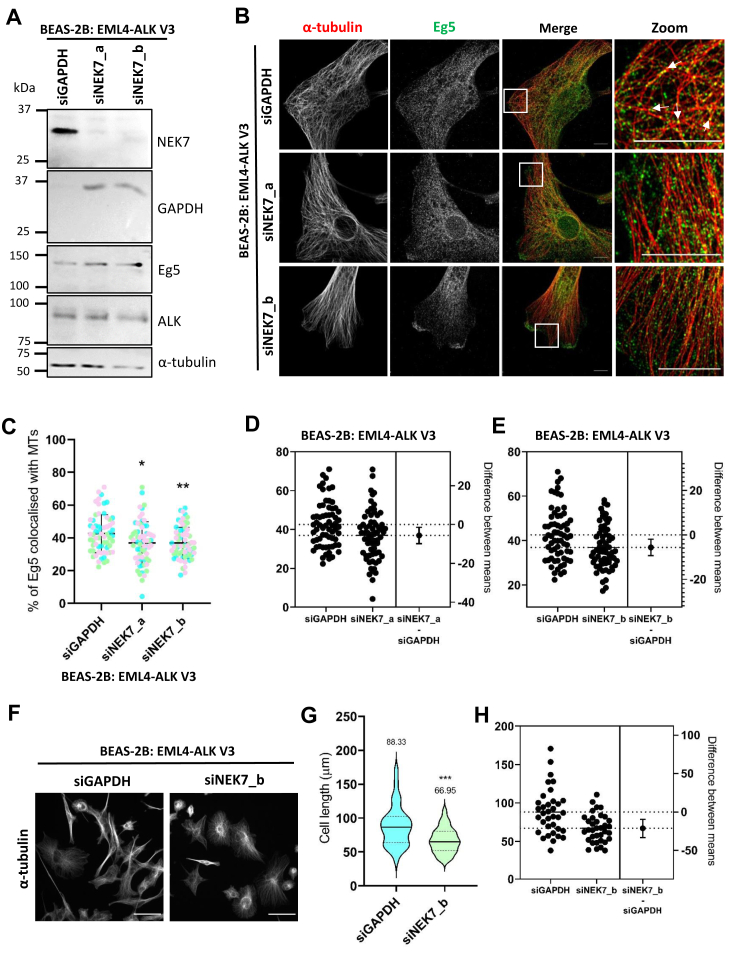
**Recruitment of Eg5 to interphase microtubules in EML4–ALK V3 cells is reduced upon NEK7 depletion.***A*, BEAS-2B:EML4–ALK V3 cells were induced for 24 h before being treated with one of two different NEK7 siRNAs (NEK7_a or NEK7_b) or GAPDH siRNA for a further 72 h. Lysates produced from induced BEAS-2B:EML4–ALK V3 cells that were treated with siRNAs against GAPDH or NEK7 were analyzed by Western blot using the antibodies indicated. Molecular weights (in kilodalton) are indicated on the *left*, and the blot is representative of three repeats. *B*, induced BEAS-2B:EML4–ALK V3 cells were treated with the siRNAs indicated before being fixed and stained with antibodies against Eg5 (*green*) and α-tubulin (*red*). Scale bars represent 10 μm. *White boxes* indicate zoom regions. *White arrows* indicate colocalization of Eg5 with microtubules. *C*, Imaris colocalization analysis was used to measure percentage colocalization on images represented in *B*. Graph represents individual data points from three independent experiments (one color per repeat); the means and standard deviations are indicated. Statistical comparisons were performed by one-way ANOVA with post hoc Games–Howell test where ∗*p* = 0.0257 and ∗∗*p* = 0.0086. *D* and *E*, estimation plots of the data shown in *C* are indicated showing differences between means for siNEK7_a (*D*) and siNEK7_b (*E*) compared with siGAPDH. *F*, BEAS-2B:EML4–ALK V3 cells were seeded onto collagen-coated coverslips and induced for 24 h before being treated with siRNAs against GAPDH or NEK7 (siNEK7_b) for a further 72 h. Cells were then fixed and stained using an α-tubulin antibody. Scale bars represent 50 μm. *G*, cell lengths were measured for cells treated as described in *F*. Violin plots represent data from three independent experiments. The *solid line* represents the median, the *dotted lines* represent quartiles, and the mean for each group is written above each plot. Significance is shown for siNEK7 compared with the control siGAPDH where ∗∗∗*p* = 0.0007. *H*, estimation plot of the data shown in *G*. ALK, anaplastic lymphoma kinase; EML4, echinoderm microtubule-associated protein-like 4.

Eg5 was previously identified as a substrate for NEK7 with phosphorylation of Eg5 by NEK7 during mitosis resulting in recruitment of this motor protein to spindle poles (21, 24). NEK7 also phosphorylates Eg5 in noncycling neurons where it contributes to dendrite extension (27). To determine whether recruitment of Eg5 to interphase microtubules in EML4–ALK V3 cells might be dependent on NEK7, NEK7 was depleted from BEAS-2B:EML4–ALK V3 cells for 72 h using two different siRNA oligonucleotides (siNEK7_a and siNEK7_b). Western blotting confirmed efficient depletion of NEK7 in cells treated with either NEK7 siRNA when compared with cells depleted of GAPDH (Fig. 3*A*). These blots also demonstrated that NEK7 depletion did not alter expression of Eg5. Depleted cells were then analyzed by immunofluorescence microscopy with Eg5 and α-tubulin antibodies. This revealed that while punctate Eg5 staining was still detected upon NEK7 depletion, it was less obviously associated with the microtubules (Fig. 3*B*). Quantification confirmed a reduction in colocalization of Eg5 with interphase microtubules after treatment with either of the two NEK7 siRNAs (Fig. 3*C*). Estimation plots were also generated for each siNEK7 oligonucleotide compared with the control sample to show the difference between the means (Fig. 3, *D* and *E*). We found that this reduction was relatively small, which may reflect only a partial reduction of NEK7 kinase activity, or the fact that NEK7-dependent phosphorylation of Eg5 is not rapidly reversed. Nevertheless, it was significant and suggests that NEK7 does indeed contribute to the recruitment of Eg5 to interphase microtubules in EML4–ALK V3 cells.

It has previously been shown that NEK7 depletion in cells expressing EML4–ALK V3 results in a loss of cytoplasmic protrusions (16). With this in mind, we also quantified the length of our BEAS-2B:EML4–ALK V3 cells that had or had not been depleted of NEK7. In agreement with the published data, we found that cell length was significantly reduced in BEAS-2B:EML4–ALK V3 cells that had been depleted of NEK7 (Fig. 3, *F*–*H*). Taken together, these results suggest that in the absence of NEK7, BEAS-2B cells expressing EML4–ALK V3 lose not only cytoplasmic protrusions but also the ability to recruit Eg5 to interphase microtubules.

### Phosphorylation of S1033 in Eg5 regulates cell length in EML4–ALK V3 cells

**Figure 4:**
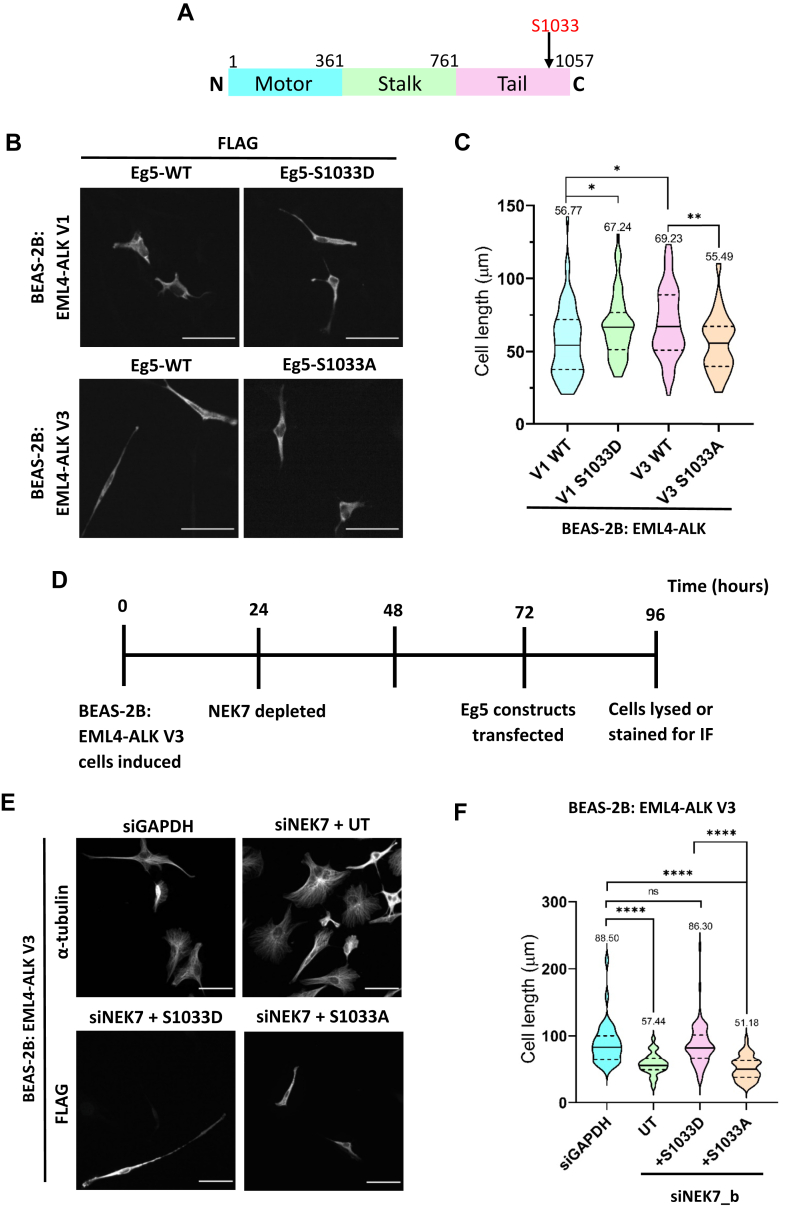
**A phosphomimetic Eg5 mutant rescues increased length of EML4–ALK V3–expressing cells depleted of NEK7.***A*, schematic representation of the Eg5 protein. Residue numbers at the junctions between domains are indicated above the schematic. The position of S1033 is also indicated. *B*, induced BEAS-2B:EML4–ALK V1 and BEAS-2B:EML4–ALK V3 cells were transfected with the indicated Eg5 constructs in the absence of an NEK7 depletion. Cells were stained with FLAG antibodies. Scale bars represent 50 μm. *C*, cell lengths were measured for cells represented in *B*. Significance is compared between groups indicated by lines on the graph. Statistical comparisons were performed by one-way ANOVA with post hoc Games–Howell testing where ∗ (V1 WT *versus* V1 S1033D) *p* = 0.0474, ∗ (V1 WT *versus* V3 WT) *p* = 0.0296, and ∗∗*p* = 0.0046. *D*, timeline of treatments performed on cells over time course of the experiment represented in *E* and *F*. *E*, induced BEAS-2B:EML4–ALK V3 cells were treated with siGAPDH or siNEK7 (siNEK7_b) oligonucleotides, and siNEK7-treated samples were cotransfected with Eg5-S1033D or Eg5-S1033A constructs or left untransfected (UT) as indicated. Cells were stained with antibodies against α-tubulin and FLAG to detect transfected cells. Scale bars represent 50 μm. *F*, cell lengths were measured for cells represented in *E*. Significance is indicated between the different groups as indicated. Statistical comparisons were performed using one-way ANOVA with post hoc Games–Howell testing where ∗∗∗∗<0.0001. In *C* and *F*, violin plots represent three independent experiments. The *solid lines* represent the medians, *dotted lines* represent quartiles, and means for each group are written above the violins. ALK, anaplastic lymphoma kinase; EML4, echinoderm microtubule-associated protein-like 4.

To more directly examine whether it is phosphorylation by NEK7 that regulates Eg5 activity in controlling cell morphology, we took advantage of the knowledge that NEK7 can phosphorylate Eg5 at S1033 (Fig. 4*A*). Phosphorylation at this site has previously been shown to be required both for recruitment of Eg5 to spindle poles in mitosis and for extension of dendrites in neurons (21, 24, 27). FLAG-tagged constructs that express either phosphonull (S1033A) or phosphomimetic (S1033D) Eg5 mutants were therefore transfected into BEAS-2B cell lines. First, BEAS-2B cells induced to express EML4–ALK V1 were seeded onto collagen-coated coverslips and were transfected with the wildtype or phosphomimetic (S1033D) Eg5 constructs. Similarly, induced BEAS-2B:EML4–ALK V3 cells were seeded in the same way and transfected with wildtype or phosphonull (S1033A) Eg5. Expression of FLAG-tagged constructs was confirmed by Western blot (Fig. S2*A*). Strikingly, a significant increase in the length of V1 cells transfected with the phosphomimetic Eg5 mutant was observed compared with those transfected with the wildtype Eg5 construct (Fig. 4, *B* and *C*). Meanwhile, cell length was significantly shorter in V3 cells transfected with the phosphonull Eg5 mutant compared with cells transfected with the wildtype construct (Fig. 4, *B* and *C*). Estimation plots confirmed the significance of these data (Fig. S2, *B* and *C*). In addition, the S1033D- or S1033A-Eg5 constructs were transfected into induced BEAS-2B:EML4–ALK V3 cells that had been depleted of endogenous NEK7 for 48 h (Fig. 4*D*). siNEK7_b was used to deplete NEK7 for these experiments as we found that this oligonucleotide gave a more consistent depletion. Mock-transfected cells that had been depleted of NEK7, as well as cells depleted of GAPDH with no further transfection, were included as controls. Cells were then analyzed by immunofluorescence microscopy with FLAG antibodies to allow identification of transfected cells and α-tubulin antibodies to enable cell morphology and length to be determined for control samples. The two stains (FLAG and α-tubulin) overlapped well, and no significant difference was found between measuring cell length with either stain (Fig. S2, *D*–*F*). In agreement with published data (16) and as demonstrated in the previous figure (Fig. 3, *F*–*H*), NEK7 depletion reduced the length of V3-expressing cells. Excitingly, expression of Eg5-S1033D, but not Eg5-S1033A, was able to rescue the cell length phenotype in NEK7-depleted cells (Fig. 4, *E* and *F*). This provides persuasive evidence that phosphorylation of Eg5 by NEK7 on S1033 regulates the morphology of BEAS-2B cells expressing EML4–ALK V3.

## Discussion

The cause of mortality in approximately 90% of cancer-related deaths is metastatic spread (37). Yet our understanding of the molecular pathways and mechanisms involved in metastasis remain far from complete. Here, we sought to better understand a pathway through which the EML4–ALK V3 fusion variant, which promotes rapid metastatic spread in NSCLC, causes cells to adopt a mesenchymal-like morphology typical of migratory and invasive tumor cells (10, 16). Previously, we had shown that EML4–ALK V3 is recruited to microtubules in interphase cells, where it likely assembles into a complex with the NEK9 and NEK7 kinases (16). Indeed, not only are NEK9 and NEK7 recruited to microtubules in cells expressing EML4–ALK V3 and required for the mesenchymal-like properties induced by EML4–ALK V3 but also expression of constitutively active mutants of NEK9 and NEK7 induces similar phenotypes in the absence of EML4–ALK V3. As NEK9 acts upstream of NEK7 (20), this led us to speculate that the morphological and migratory changes induced by EML4–ALK V3 depend on the activation of NEK7 on microtubules of interphase cells.

**Figure 5:**
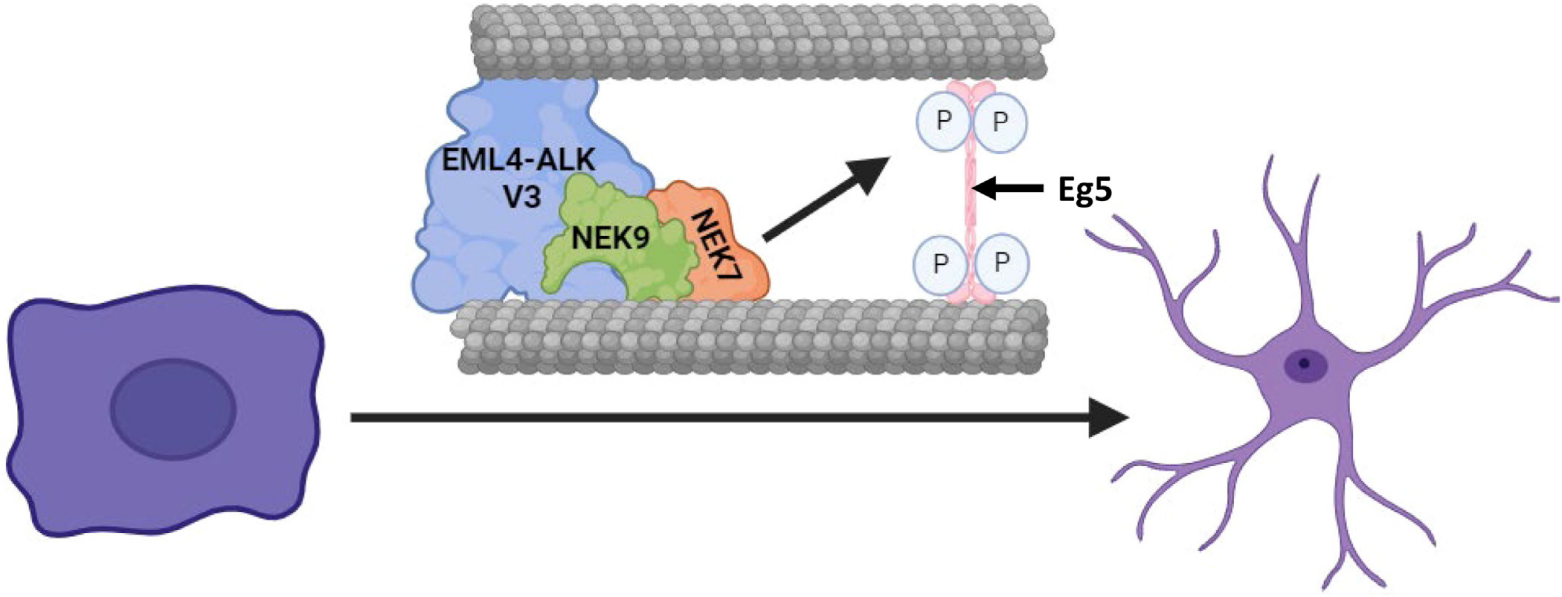
**Phosphorylation of Eg5 occurs downstream of the EML4–ALK V3–NEK9–NEK7 complex.** The schematic model illustrates the presence of a complex consisting of EML4–ALK V3 (*blue*), NEK9 (*green*), and NEK7 (*orange*) associated with interphase microtubules. Association with EML4–ALK V3 leads to activation of NEK9, which in turn activates NEK7. Active NEK7 then phosphorylates each Eg5 monomer (*pink*) on S1033 causing recruitment of the Eg5 tetramer to interphase microtubules. This contributes to development of an elongated cell morphology where long protrusions form potentially as a result of crosslinking or bundling of interphase microtubules by Eg5. ALK, anaplastic lymphoma kinase; EML4, echinoderm microtubule-associated protein-like 4.

In dividing cells, NEK9 and NEK7 reach maximal activity in mitosis downstream of CDK1 and PLK1 (19, 24, 38). Here, they contribute to mitotic spindle assembly through a variety of mechanisms. One key substrate of NEK7 that is required for bipolar spindle assembly is the kinesin motor, Eg5 (21, 24). In most cells, Eg5 is only recruited to microtubules in mitosis as a result of its phosphorylation by not only NEK7 but also CDK1. Intriguingly, we discovered that expression of EML4–ALK V3 led to the recruitment of Eg5 to microtubules during interphase in dividing cells. Furthermore, chemical inhibitors of Eg5 reduced the morphological changes normally seen upon expression of EML4–ALK V3, whereas depletion of NEK7 and expression of Eg5 phosphomutants strongly suggest that these phenotypes are likely to depend upon phosphorylation of Eg5 by NEK7 (Fig. 5).

The mesenchymal-like morphology of cells expressing EML4–ALK V3 is associated with formation of elongated cytoplasmic protrusions that contain both microtubules and actin (16). Our experiments revealed not only that Eg5 contributes to the change of cell morphology but also that these changes are dependent on Eg5 motor activity. Specifically, these changes were lost when cells expressing EML4–ALK V3 were treated with an L5 inhibitor that weakens microtubule binding of Eg5 and with a rigor inhibitor that locks Eg5 onto microtubules. This indicates that the presence of Eg5 on interphase microtubules alone is not sufficient for cytoplasmic protrusions to form, but that Eg5 must be capable of plus end–directed movement. Moreover, it suggests that the microtubule crosslinking and bundling activity of Eg5 is likely to contribute to the formation of cytoplasmic extensions. Indeed, cells expressing EML4–ALK V3 also exhibit excessive microtubule stabilization, which may be a result of the increased microtubule bundling. Interestingly though, besides crosslinking adjacent microtubules, Eg5 has polymerase activity at microtubule plus ends (28, 29). It is therefore plausible that formation of cytoplasmic protrusions results from increased growth of microtubules that causes the cell membrane to be pushed forward.

Importantly, our results suggest that recruitment of Eg5 to interphase microtubules in cells expressing EML4–ALK V3 depends upon phosphorylation of Eg5 on S1033. This is the site phosphorylated by NEK6 and NEK7 that leads to recruitment of Eg5 to centrosomes in mitotic cells (21, 24). However, this site in Eg5 is also phosphorylated by NEK7 in nondividing neurons (27). Indeed, transfection of an Eg5-S1033D phosphomimetic mutant, but not the wildtype Eg5 protein or an Eg5-S1033A phosphonull mutant, could rescue the length of neurites depleted of NEK7 (27). In cells expressing EML4–ALK V3, depletion of NEK7 led to reduced colocalization of Eg5 with interphase microtubules. Moreover, expression of the phosphomimetic Eg5-S1033D mutant was also able to restore the elongated cell morphology following depletion of NEK7, whereas a phosphonull Eg5-S1033A mutant could not. Additionally, expression of the phosphomimetic Eg5 mutant also increased the length of cells expressing EML4–ALK V1, whereas the phosphonull mutant reduced the length of cells expressing EML4–ALK V3 that had not been depleted of NEK7. Given that expression of the phosphonull Eg5 mutant led to reduced length of V3 cells in the absence of an NEK7 depletion, we believe this mutant likely has a dominant negative effect through incorporation with endogenous Eg5 into tetrameric Eg5 motor proteins. We therefore conclude that phosphorylation of Eg5 by NEK7 is an important step in the creation of a mesenchymal-like morphology in cells expressing EML4–ALK V3. Interestingly, generation of a phosphospecific antibody directed against pS1033 would enable the phosphorylation state of Eg5 in EML4–ALK cell lines and tumors to be analyzed. Future studies should also seek to further understand whether phosphorylation of Eg5 by NEK7 is contributing to microtubule stabilization and enhanced cell migration in cells expressing EML4–ALK V3.

Following discovery of the EML4–ALK fusion protein, first-line treatment of NSCLC patients with ALK+ tumors has entailed the use of targeted ALK inhibitors, with crizotinib, the first approved inhibitor in 2011 (39). Second- and third-generation ALK inhibitors have since been approved that overcome some of the on-target resistance mechanisms that develop against first-generation inhibitors like crizotinib. However, there remains an issue of acquired resistance and, in trials undertaken to date, patients with EML4–ALK V3 do not respond as well to these targeted ALK inhibitors (9, 10). This could be in part because of activation of pathways that are independent of ALK activity, such as the one formed by EML4–ALK V3–NEK9–NEK7 and its downstream effectors. Our finding that Eg5 is a downstream target of this signaling module that contributes to the development of mesenchymal-like cell morphology identifies a potential novel target for future treatment of these patients. Considerable efforts have been put into developing potent and selective Eg5 inhibitors that show clinical efficacy. However, to date, filanesib is the only Eg5 inhibitor to progress to phase III clinical trials, where it has shown promising results in the treatment of multiple myeloma (40, 41). It will be exciting to investigate whether Eg5 inhibitors could be used in the treatment of EML4–ALK+ NSCLC either alone or in combination with ALK inhibitors. Moreover, EML4–ALK fusions have been identified in a number of other cancers, including breast, colorectal, and pancreatic cancers, so these findings may be more widely applicable to these cancer types (5, 6, 7).

## Experimental procedures

### Plasmids

pCS2-FLAG-Eg5 S1033D and S1033A constructs were previously described (27).

### Cell culture, drug treatments, and transfection

BEAS-2B parental cells were cultured in RPMI1640 medium (Invitrogen) supplemented with 10% fetal bovine serum, 100 μg/ml penicillin—streptomycin, and 100 μg/ml G418. BEAS-2B:EML4–ALK V1 and V3 stable cell lines were cultured as for the parental cells with the addition of 0.25 μg/ml puromycin. Expression of EML4–ALK V1 or V3 in BEAS-2B cell lines was induced using 1 μg/ml doxycycline for 72 h, unless otherwise stated. H3122 (V1) and H2228 (V3) cells were cultured using RPMI1640 medium supplemented with 10% fetal bovine serum and 100 μg/ml penicillin–streptomycin. All cells were cultured at 37 °C with 5% CO_2_ atmosphere. H3122 (V1) and H2228 (V3) cells were obtained from American Type Culture Collection within the last 10 years, whereas BEAS-2B parental and derived cell lines were received from Dr Jene Choi’s laboratory. All cells were stored in liquid nitrogen and kept in culture for a maximum of 2 months. An in-house PCR-based system was used to test for mycoplasma infection every 2 months, and cells were free from mycoplasma infection in all experiments undertaken. Collagen-coated coverslips were prepared by pipetting 1 mg/ml rat tail (type I) collagen (Corning) on acid-etched glass coverslips and incubating for 1 h. Coverslips were then washed three times with PBS and left to dry for 10 min. Collagen embedding of cells was performed as described (42). Briefly, collagen was prepared by neutralizing a stock solution with 10× Dulbecco's modified Eagle's medium and reconstitution buffer (42). Once neutralized, a layer of collagen was added to a chamber slide and polymerized at 37 °C, before cell suspension was added to allow cells to adhere to the collagen layer. Cells were allowed to adhere for 30 min at 37 °C before a second layer of collagen was added on top of the cells and polymerized at 37 °C. Once the top layer of the collagen had polymerized, cell culture media were added to the chamber slides, and cells were left to spread for 24 h. Where indicated, cells were treated with 100 nM filanesib (Generon) or 10 μM BRD9876 (Generon). Both drugs were reconstituted in dimethyl sulfoxide. Transient transfections were performed using FuGENE HD (Promega) according to the manufacturer’s instructions.

### Preparation of cell extracts, SDS-PAGE, and Western blotting

Cells were lysed in radioimmunoprecipitation assay buffer (50 mM Tris–HCl [pH 8], 150 mM NaCl, 1% SDS, 0.5% sodium deoxycholate, 0.5% Nonidet P-40, 0.5% Triton X-100, supplemented with 1:100 RNAse A, 1:1000 DNAse, 1:100 DTT, and 1:1000 Protease Inhibitor Cocktail) prior to analysis by SDS-PAGE and Western blotting. Primary antibodies used were ALK (Cell Signaling [catalog no.: D5F3], 1:1000 dilution), α-tubulin (Invitrogen [catalog no.: PA5-19489], 1:5000 dilution), GAPDH (Cell Signaling [catalog no.: 14C10], 1:500 dilution), and NEK7 (Cell Signaling [catalog no.: C34C3], 1:1000 dilution). Secondary antibodies used were horseradish peroxidase–conjugated antimouse or anti-rabbit antibodies (Bethyl Laboratories [catalog nos.: A90-116P and A120-101P, respectively], 1:2000 dilution). Western blots were developed using an enhanced chemiluminescence kit (Pierce) and exposed to X-ray film. Quantification of Western blots was performed in Fiji by measuring band intensity and normalizing to the relevant loading control.

### RNAi

Cells were seeded at 30 to 40% confluency in Opti-MEM Reduced Serum Medium (Invitrogen) and transfected with 50 nM siRNA duplexes using Oligofectamine (Invitrogen), according to the manufacturer's instructions. At 72 h after transfection, cells were either fixed for immunofluorescence staining or prepared for Western blotting. siRNA oligos were directed against NEK7 (Dharmacon [catalog nos.: J-003795-12-0050 and J-003795-14-0050]) or GAPDH (Ambion [catalog no.: AM4631]).

### Fixed and time-lapse microscopy

Cells grown on acid-etched glass coverslips were fixed and permeabilized by incubation in ice-cold methanol at −20 °C for a minimum of 20 min. Cells were blocked with PBS supplemented with 3% bovine serum albumin and 0.2% Triton X-100 prior to incubation with the appropriate primary antibody diluted in PBS supplemented with 3% bovine serum albumin and 0.2% Triton X-100. Primary antibodies used were Eg5 (Proteintech [catalog no.: 23333-1-AP], 1:200 dilution), α-tubulin (Sigma [catalog no.: T5168], 1:1000 dilution), and FLAG (Sigma [catalog no.: F7425], 1:500 dilution). Secondary antibodies were Alexa Fluor 488-, 594-, and 647-conjugated anti-rabbit or antimouse antibodies (Invitrogen, all 1:200 dilution). For super-resolution microscopy, single optical sections were acquired using a Zeiss LSM980 Airyscan 2 microscope fitted with a Plan-Apochromat 63× objective (numerical aperture [NA] = 1.4). For acquisition of lower resolution confocal images, single optical sections were imaged using a VisiTech Infinity 3 confocal microscope fitted with a Hamamatsu C11440-22CU Flash 4.0 V2 sCMOS camera, and a Plan Apo 60× objective (NA = 1.4) or Plan Apo 20× objective (NA = 0.75) was used. For live cell imaging, a PhaseFocus LiveCyte 2 label-free imaging system fitted with a PLN 10× objective (NA = 0.25) was used. Cell lengths were measured in Fiji (36), where a line was drawn across the longest length of the cell, through the nucleus.

### Imaris colocalization analysis

Colocalization was analyzed from two channel super-resolution images using Imaris software. Briefly, a mask was drawn in Fiji over an area of the cytoplasm where distinct microtubules were visible and overlayed in Imaris creating a designated region of interest. Images were then thresholded, and a separate channel was built to measure colocalization of pixels between the two channels in this region of interest (43). Statistics were obtained for this channel, including the percentage of channel A colocalized with channel B.

### Statistical analysis

Data were analyzed in GraphPad Prism, version 9 (GraphPad Software, Inc). Scatter plots show individual data points from three independent experiments (each color represents one repeat), together with means and standard deviations. Violin plots show the spread of data from three independent experiments, where the solid center line represents the median, dotted lines represent quartiles, and the numbers written above each violin represent the mean of that group. Estimation plots show the difference between the means and were generated automatically by GraphPad Prism when Student’s *t* test was performed. Statistical analysis was also performed in GraphPad Prism 9. Student’s unpaired *t* test assuming unequal standard variation was used to compare means from two groups, whereas one-way ANOVA with post hoc tests was used to compare means of multiple groups. *p* Values represent ∗*p* < 0.05, ∗∗*p* < 0.01, ∗∗∗*p* < 0.001, ∗∗∗∗*p* < 0.0001, and ns, not significant.

## Data availability

All data described are contained within this article or the supporting information.

## Supporting information

This article contains supporting information.

## Conflict of interest

The authors declare that they have no conflicts of interest with the contents of this article.
