# Supplementary material for: The mesenchymal morphology of cells expressing the EML4–ALK V3 oncogene is dependent on phosphorylation of Eg5 by NEK7
Source: J Biol Chem. 2024 Mar 6;300(5):107144. doi: 10.1016/j.jbc.2024.107144 (PMC11061729; doi:10.1016/j.jbc.2024.107144)
Supplement: Supporting information [file mmc1.pdf]

SUPPLEMENTARY INFORMATION

Supplementary Figure S1. Eg5 inhibitors arrest cells in mitosis with monopolar spindles

A Induced BEAS-2B:EML4-ALK V1 (A) or V3 (B) cells were embedded into collagen and treated with DMSO or the Eg5 inhibitors, Filanesib (100 nM) or BRD9876 (10 μM) for 24 hours and examined by live cell imaging. **B & C**. The percentage of cells in mitosis was quantified for V1 cells (B) and V3 cells (C). Graphs represent three independent experiments and show the means and standard deviations. Significance is determined in comparison to the DMSO treated cells. Statistical comparisons were performed by one way ANOVA with post-hoc Dunnett's T3 test where in (B) **** $P < 0.0001$ and ** $P = 0.0014$ and in (C) ** $P = 0.0021$ and * $P = 0.0155$. **D**. Induced BEAS-2B:EML4-ALK V3 cells were treated with Eg5 inhibitors for 24 hours before being stained with antibodies against α-tubulin (red) and γ-tubulin (green); DNA was stained with Hoechst 33258 (blue). Scale bars, 5 μm.

Supplementary Figure S2. Expression of FLAG-Eg5 constructs in BEAS-2B:EML4-ALK cell lines

A. BEAS-2B cells were induced for 48 hours before being transfected with the indicated FLAG-Eg5 constructs for 24 hours. WT, WT-Eg5; SD, S1033D-Eg5; SA, S1033A-Eg5. Lysates were produced and analysed by Western blot using the indicated antibodies. Molecular weights (kDa) are indicated on the left. **B & C.** Estimation plots of the data shown in Fig 4C showing the differences between the means of V1 WT and V1 S1033D (B) and V3 WT and V3 S1033A (C). **D.** Induced BEAS-2B:EML4-ALK V3 cells were depleted of NEK7 for 48 hours before being transfected with the Eg5 phosphomutant constructs. Cells were stained with FLAG and α-tubulin antibodies and cell lengths (indicated by white lines) measured using both stains. Scale bars, 50 μm. **E & F.** Cell lengths were measured for cells treated as described in D. Statistical comparisons were made using students t-test where in E, n.s = 0.7425 and in F, n.s. = 0.9527. Graphs show means and standard deviations from two independent experiments.

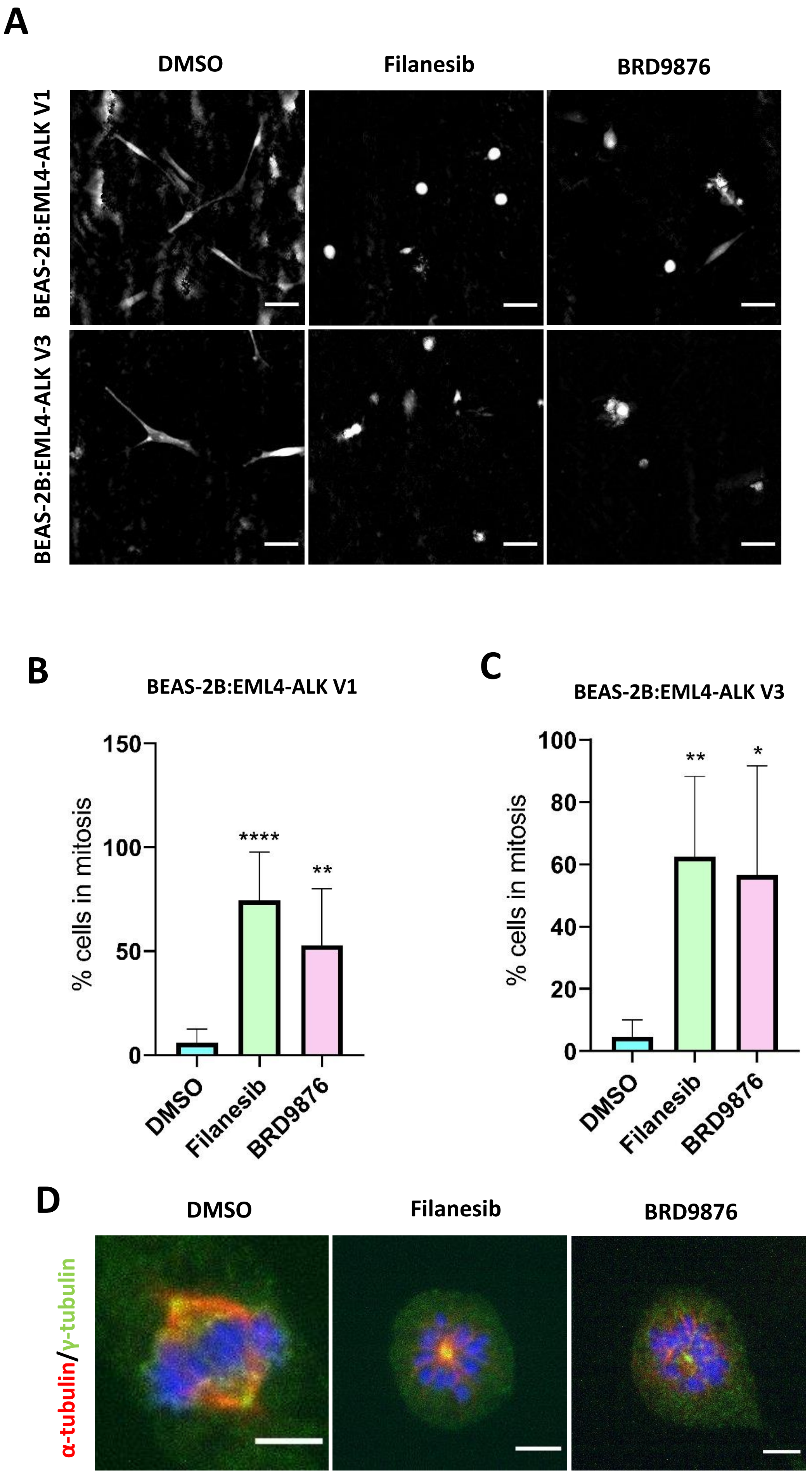
A
DMSO
Filanesib
BRD9876
BEAS-2B:EML4-ALK V1
BEAS-2B:EML4-ALK V3
B
BEAS-2B:EML4-ALK V1
% cells in mitosis
150
100
50
0
****
**
DMSO
Filanesib
BRD9876
C
BEAS-2B:EML4-ALK V3
% cells in mitosis
100
80
60
40
20
0
**
*
DMSO
Filanesib
BRD9876
D
DMSO
Filanesib
BRD9876
α-tubulin/γ-tubulin

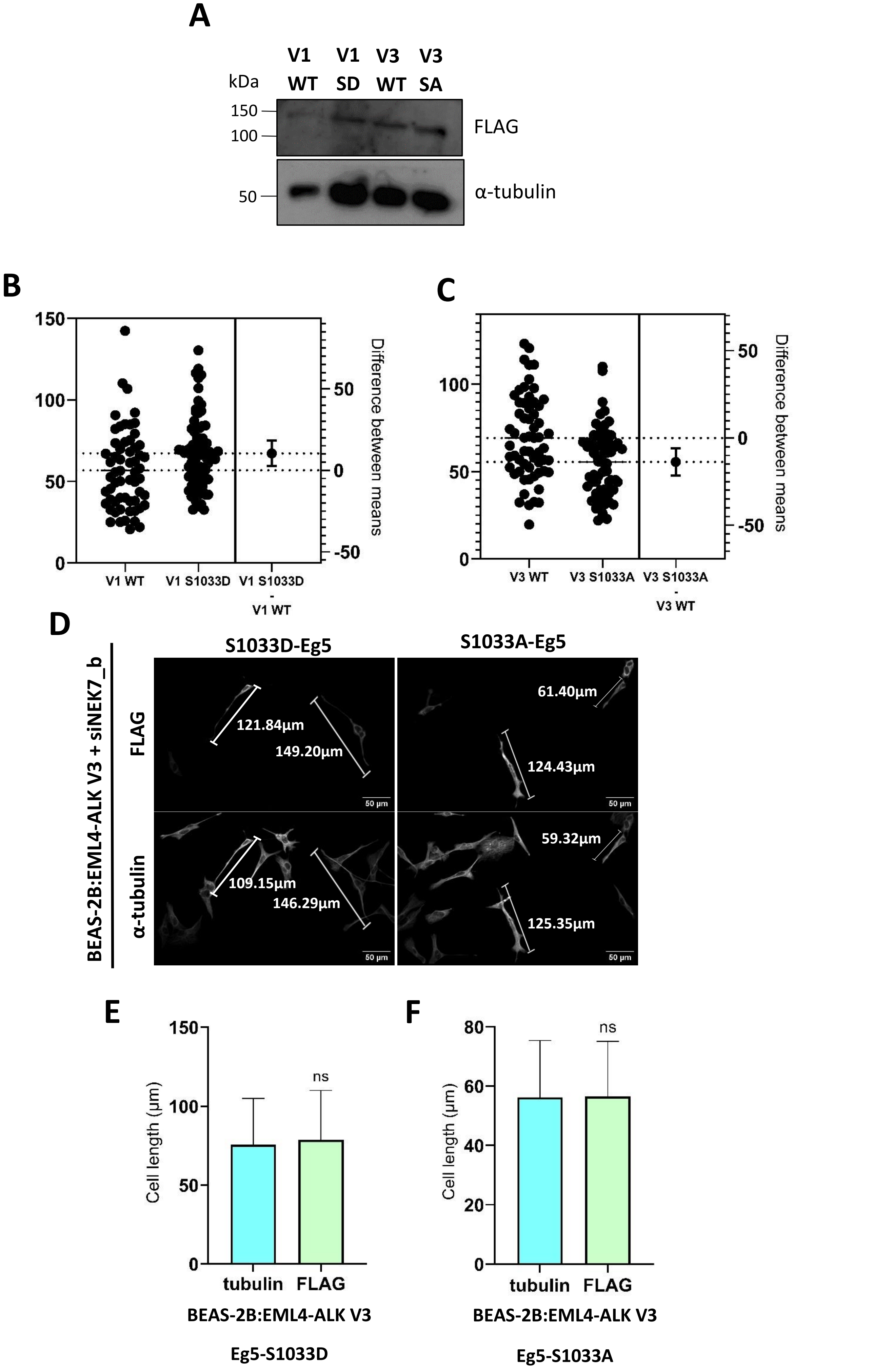
A
V1 WT
V1 SD
V3 WT
V3 SA
kDa
150
100
50
FLAG
α-tubulin
B
150
100
50
0
Difference between means
50
0
-50
V1 WT
V1 S1033D
V1 S1033D - V1 WT
C
100
50
0
50
0
-50
Difference between means
V3 WT
V3 S1033A
V3 S1033A - V3 WT
D
BEAS-2B:EML4-ALK V3 + siNEK7_b
S1033D-Eg5
S1033A-Eg5
FLAG
α-tubulin
121.84µm
149.20µm
61.40µm
124.43µm
109.15µm
146.29µm
59.32µm
125.35µm
50 µm
E
150
100
50
0
Cell length (µm)
ns
tubulin
FLAG
BEAS-2B:EML4-ALK V3
Eg5-S1033D
F
80
60
40
20
0
Cell length (µm)
ns
tubulin
FLAG
BEAS-2B:EML4-ALK V3
Eg5-S1033A
